# Magnetic Particle Imaging Reveals that Iron-Labeled Extracellular Vesicles Accumulate in Brains of Mice with Metastases

**DOI:** 10.1021/acsami.4c04920

**Published:** 2024-06-11

**Authors:** Victoria A. Toomajian, Anthony Tundo, Evran E. Ural, Emily M. Greeson, Christopher H. Contag, Ashley V. Makela

**Affiliations:** † Institute for Quantitative Health Science and Engineering, Michigan State University, East Lansing, Michigan 48824, United States; ‡ Department of Biomedical Engineering, Michigan State University, East Lansing, Michigan 48824, United States; § Department of Microbiology, Genetics & Immunology, Michigan State University, East Lansing, Michigan 48824, United States

**Keywords:** breast cancer, magnetic particle imaging, exosome, extracellular vesicles, brain metastasis

## Abstract

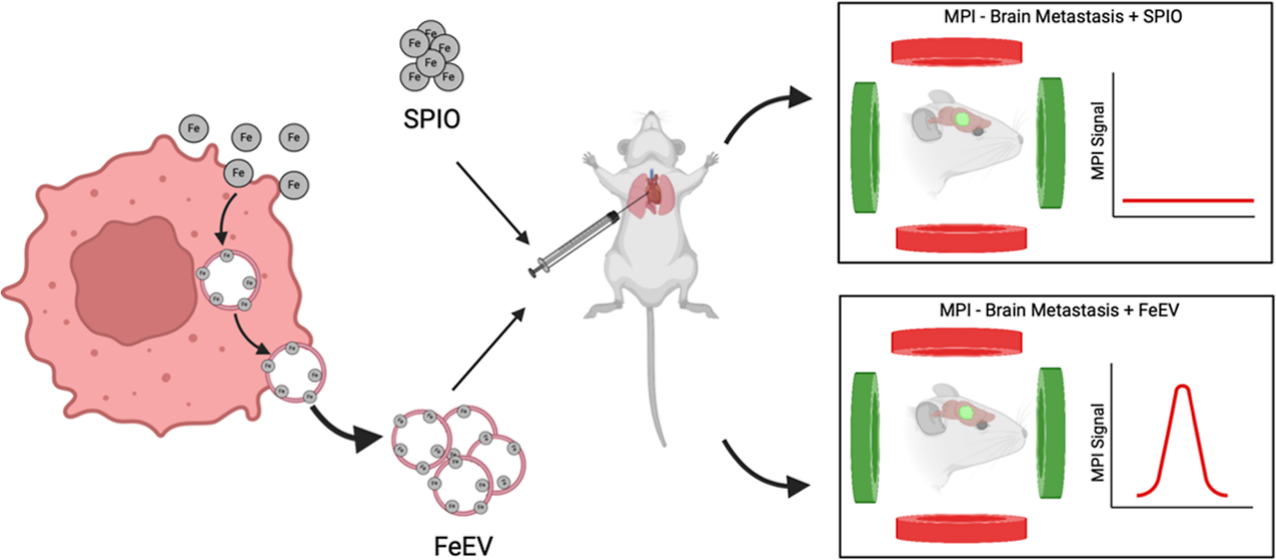

The incidence of breast cancer remains high worldwide and is associated with a significant risk of metastasis to the brain that can be fatal; this is due, in part, to the inability of therapeutics to cross the blood–brain barrier (BBB). Extracellular vesicles (EVs) have been found to cross the BBB and further have been used to deliver drugs to tumors. EVs from different cell types appear to have different patterns of accumulation and retention as well as the efficiency of bioactive cargo delivery to recipient cells in the body. Engineering EVs as delivery tools to treat brain metastases, therefore, will require an understanding of the timing of EV accumulation and their localization relative to metastatic sites. Magnetic particle imaging (MPI) is a sensitive and quantitative imaging method that directly detects superparamagnetic iron. Here, we demonstrate MPI as a novel tool to characterize EV biodistribution in metastatic disease after labeling EVs with superparamagnetic iron oxide (SPIO) nanoparticles. Iron-labeled EVs (FeEVs) were collected from iron-labeled parental primary 4T1 tumor cells and brain-seeking 4T1BR5 cells, followed by injection into the mice with orthotopic tumors or brain metastases. MPI quantification revealed that FeEVs were retained for longer in orthotopic mammary carcinomas compared to SPIOs. MPI signal due to iron could only be detected in brains of mice bearing brain metastases after injection of FeEVs, but not SPIOs, or FeEVs when mice did not have brain metastases. These findings indicate the potential use of EVs as a therapeutic delivery tool in primary and metastatic tumors.

## Introduction

Among all new cases of cancer worldwide in 2022, the incidence of breast cancer is the highest in females at 23.8%.^1^ Although there have been advances in treating the primary tumor, metastases continue to be the leading cause of death among these patients.^2^ In particular, up to 30% of breast cancer patients have brain metastases,^2,3^ and the prognosis for these patients is poor with a survival rate of only 20% at 1 year.^3−6^

Treatment of brain metastasis includes radiotherapy and surgery, and the use of these options is dependent on the amount of metastatic burden in the brain. However, these have limited survival benefit and can result in a reduced quality of life.^7,8^ Systemic treatments such as cytotoxic chemotherapy may be used, but these therapies have difficulties reaching the tumor due to the presence of the blood–brain barrier (BBB) which is formed by endothelial cells and tight junctions.^9,10^ When the BBB remains intact, the use of a systemic treatment will not reach the target and will not elicit any therapeutic outcome. However, the BBB can be disrupted in a heterogeneous manner with the formation of a tumor, resulting in what is known as the blood–tumor barrier (BTB).^9,10^ This heterogeneous permeability causes changes in accumulation of chemotherapies; this has been observed in experimental models of brain metastases where there has been evidence of impairment of the BTB, although some barrier functions remain intact, limiting the accumulation of drugs to such low amounts which do not elicit any therapeutic effect.^11,12^ A strategy for improving the delivery of therapeutics across these barriers is needed to effectively combat brain metastases.

One possibility is to use naturally derived particles with lipid membranes to deliver therapeutics across the BBB or improve delivery across the BTB. Extracellular vesicles (EVs) are small particles naturally released from cells that carry and deliver bioactive molecules as a method of cell-to-cell communication, and EVs from various types of cells have been shown to target tumors and cross the BBB.^13,14^ EVs can be classified by mechanism of biogenesis, size, function, or composition; examples of EVs classified by biogenesis include exosomes, microvesicles, and apoptotic bodies.^15^ Exosomes are formed by internal budding within a multivesicular body and range in size from 40 to 150 nm in diameter. Microvesicles are formed by an external budding of the cell membrane and are typically 100 nm to 1 μm in diameter. Apoptotic bodies, the largest class of vesicles, are released from dead and dying cells and can be up to several micrometers in diameter. Because of the overlapping size range of microvesicles and exosomes, EVs are often characterized as enriched populations of small EVs and large EVs rather than pure populations.^15^ Because EVs can circulate in the bloodstream for a prolonged period of time, can cross the BBB,^13,14,16−21^ and can be manipulated as carriers of drugs, nucleic acids, or nanoparticles,^18−30^ there is vast potential to engineer EVs as imaging or delivery tools to target brain metastases. Previous studies have labeled EVs with superparamagnetic iron oxide (SPIO) nanoparticles for in vivo tracking using magnetic resonance imaging (MRI)^25−27,31,32^ and to a lesser extent, magnetic particle imaging (MPI).^33^ Additionally, EVs derived from tumor cells have been shown to improve delivery to parental tumor cells in vivo.^34−36^ A recent study has used photoacoustic imaging to detect Prussian blue nanoparticles within an orthotopic brain tumor model in mice.^37^ Coating the particles with glioma U-87 derived EVs facilitated the delivery of the Prussian blue nanoparticles into the tumor region within the brain.

An imaging technique that could facilitate the improvement of EV delivery across the BBB or BTB would offer significant benefits for treating patients suffering from brain metastases. Identifying localization and accumulation in the region of interest would provide confirmation of successful delivery and assess retention over time. MPI is a sensitive and quantitative imaging method that detects the magnetic properties of SPIOs directly^38−40^ and offers the opportunity to assess the biodistribution of EVs associated with SPIOs. MPI benefits from essentially no background magnetic signals in tissue with signals originating only from SPIOs. In addition, there is no loss of signal due to mammalian tissues, and thus the depth of particles within the body does not adversely affect quantitative imaging. MPI has been used for cancer detection^38,41−44^ and has been used to track engineered EVs to primary tumors^33^ in animal models. In addition to this, other applications of MPI have included vascular imaging,^45−49^ inflammation,^50,51^ therapeutic studies^52,53^ and cell tracking.^53−59^ Further, groups are working toward improved nanoparticles tailored for MPI,^60−64^ improvements in MPI hardware and acquisition^65,66^ and image analysis.^67,68^ Other imaging methods such as in vivo bioluminescence imaging (BLI) can be used as a complementary imaging modality in rodent models to associate or correlate MPI signals with other biological processes such as sites of metastatic lesions.^69^

In this study, we labeled murine breast cancer cells and a brain seeking derivative cell line (4T1 and 4T1BR5, respectively) with SPIOs, which led to the production of iron-labeled EVs (FeEVs) in the culture medium. The FeEVs were tracked using MPI to monitor their retention in a primary breast tumor or accumulation in the heads of mice that had brain metastasis.

## Results and Discussion

### Labeling Cells with Iron Leads to Production of Iron-Labeled EVs

**Figure 1:**
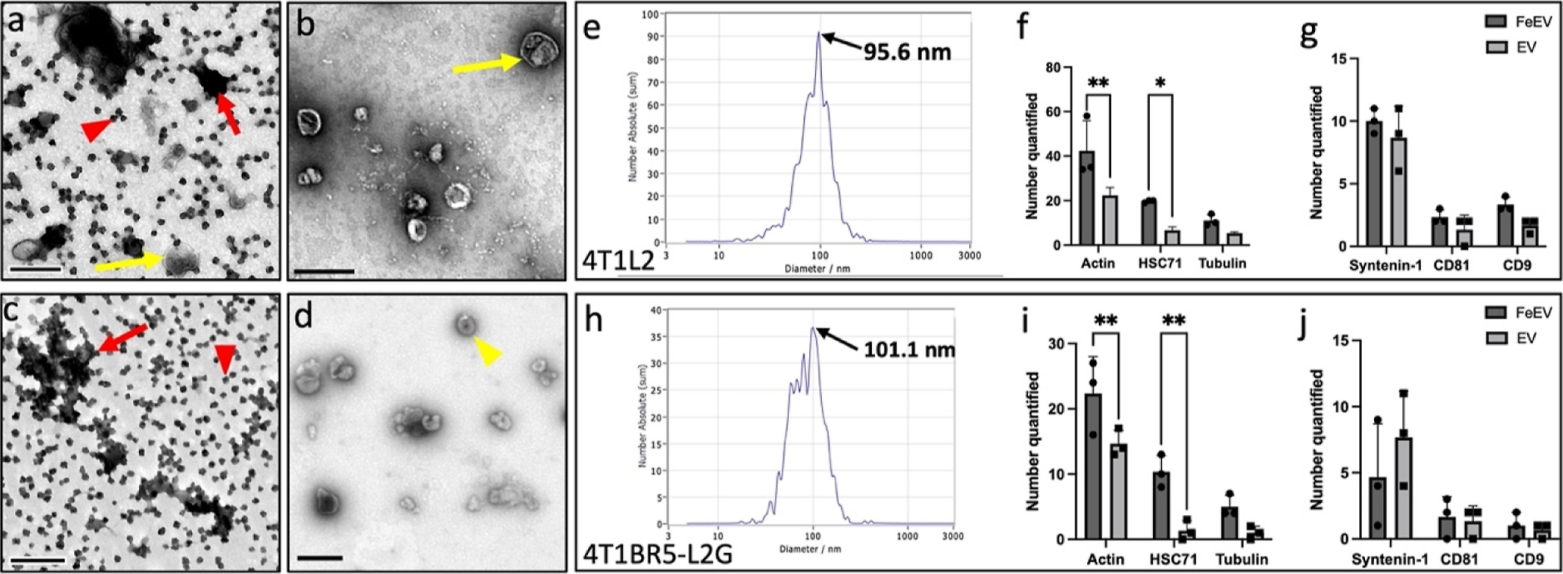
Iron-labeled EV characterization. Iron-labeled EVs (FeEVs) (a,c, 4T1L2 and 4T1BR5-L2G, respectively) and unlabeled EVs (b,d, 4T1L2 and 4T1BR5-L2G, respectively) were visualized via TEM. Both free iron (red arrowheads) and EVs associated with iron (red arrows) can be seen in the FeEV samples. FeEVs and EVs both display the classical cup shape (yellow arrows) and round shape (yellow arrowhead). Nanoparticle tracking analysis determined the peak FeEV size in 4T1L2 (e) and 4T1BR5-L2G (h) cells. Abundance of cytosolic and membrane proteins typically associated with EVs were quantified in 4T1L2 (f,g) and 4T1BR5-L2G (i,j) EVs and FeEVs as detected by mass spectrometry. Scale bars = 250 nm. *p < 0.05, **p < 0.01.

Coincubation of 4T1-fLuc2 (4T1L2) or 4T1BR5-fLuc2/GFP (4T1BR5-L2G) cells in culture with SPIO nanoparticles resulted in the cell secretion of iron-labeled EVs (FeEVs) into the culture medium. This method of protamine sulfate/heparin assemblies to aid in Synomag-D internalization into 4T1BR5 cells has been performed previously, with no changes in viability of cells up to 3 days postlabeling.^41^ Upon isolation from conditioned medium via differential centrifugation, the SPIOs were found to be associated with the cell-secreted EVs, as confirmed using transmission electron microscopy (TEM) visualizing the EV membranes (stained with uranyl acetate for contrast) and our dextran-coated iron nanoparticles. Free iron particles (red arrowheads) and iron particles associated with EVs (red arrows) were observed in the samples (Figure 1a,c; 4T1L2 and 4T1BR5-L2G, respectively). Images showed iron to be associated with the EV membrane; it did not appear that the EV membranes encapsulated the SPIOs. After the sample was washed, a large amount of what appeared to be free iron particles was still present in the preparations. The FeEVs and EVs from cells which were not cultured with iron (Figure 1b,d; 4T1L2 and 4T1BR5-L2G, respectively) appeared to have a similar size and morphology, both appearing round (yellow arrowheads) and often having the classical cup shape (yellow arrow).

Enriched FeEV samples were characterized by examining the particle size and composition with regard to the iron content and proteins. Iron content per EV was quantified by imaging different masses of Synomag-D by MPI (Figure S1a). The linear relationship between iron mass and MPI signal (Figure S1b, *R*^2^ = 0.996), allows for a quantitative measure of iron (Table S1) in the FeEV pellets imaged by MPI (Figure S1c). The average peak size of the 4T1L2 cell-derived FeEVs was 98.8 ± 4.98 nm (Figure 1e, representative image) and 98.43 ± 13.79 nm in 4T1BR5-L2G cell-derived FeEVs (Figure 1h, representative image). These sizes are within the average size of small EVs, typically defined as 30–150 nm,^70^ but larger than the 70 nm native SPIO particles. The FeEVs contained similar iron loadings, with 0.57 fg of iron per EV (Fe/EV) and 0.51 fg of Fe/EV in 4T1L2 and 4T1BR5-L2G FeEVs, respectively. Based on a lower MPI detection limit of ∼313 ng of Synomag-D (in 1 μL), using our standard imaging parameters (Figure S1a), we could expect a sensitivity of 5.52–7.42 × 10^8^ FeEVs, using the average Fe/EV for 4T1BGL, 4T1L2 and 4T1BR5 derived FeEVs. However, the volume in which the FeEVs accumulate may vary, resulting in a sensitivity higher than or lower than our prediction.

Mass spectrometry analysis of FeEVs and EVs derived from 4T1L2 (Figure 1f,g) and 4T1BR5-L2G (Figure 1i,j) cells showed expression of EV-associated cytoskeletal proteins actin and tubulin α-1A, known to be promiscuously incorporated in EVs.^70^ Further, heat shock cognate 71 (HSC71), tetraspanins CD81 and CD9, and Syntenin-1 were also identified in the samples. FeEVs contained significantly more actin and HSC71 in both 4T1L2 (*p* = 0.001 and *p* = 0.02, respectively) and 4T1BR5-L2G (*p* = 0.007 and *p* = 0.002, respectively) when compared to EVs without iron. Actin and HSC71 have been identified as phagosomal proteins,^71,72^ so the increased expression observed in FeEVs may be due to phagocytic activity in cells upon coincubation with iron, and promiscuous incorporation into the secreted FeEVs.

There was no difference in tubulin α-1A expression between FeEVs and EVs for either 4T1L2 or 4T1BR5-L2G cells (*p* = 0.26 and *p* = 0.113, respectively). No differences were found in expression of syntenin-1, CD81, and CD9 between FeEVs and EVs in 4T1L2 (*p* = 0.223, *p* = 0.354 and *p* = 0.134, respectively) and 4T1BR5-L2G cells (*p* = 0.147, *p* = 0.866 and *p* = 0.866, respectively). Overall, cellular incorporation of iron into EVs did not affect expression of typical EV markers involved in EV biogenesis and release, which has been observed previously.^73^

There were some proteins found in EVs and FeEVs derived from 4T1BR5-L2G cells that were not present in those derived from 4T1L2 cells, highlighting the brain-seeking metastatic nature of the 4T1BR5-L2G EVs. Osteopontin (OPN), a protein ligand of CD44 and considered a metastasis gene, was only found in the 4T1BR5-L2G EVs and FeEVs but was not present in 4T1L2 EVs or FeEVs; OPN is produced by tumor cells and increased levels have been associated with tumor cell migration and metastasis in vivo,^74^ brain tumors,^75,76^ and OPN has a role in BBB function.^77,78^ Further, Semaphorin 3E (Sema3E) was found only in 4T1BR5-L2G FeEVs but not 4T1L2 FeEVs. Sema3E is expressed in vasculature and many areas of the nervous system including the brain,^79,80^ has been shown to be highly expressed in metastatic cancer cells^81^ and is associated with cancer cell invasiveness, migration and metastases in vivo.^82^ Interestingly, in a separate study, a Sema3E knockdown model only affected metastatic growth, but not the growth of the primary tumor,^81^ emphasizing the role of Sema3E in metastasis. Additional proteins only found in the 4T1BR5-L2G EVs/FeEVs were (1) Netrin-1, which plays a role in BBB functions^83^ and has been associated with glioblastoma;^84,85^ (2) C–X–C motif chemokine 5 (CXCL5), which is secreted by microglia in brain metastasis,^86^ and is upregulated in breast cancer metastasis;^87^ and (3) galectin-3, which has been implicated in breast cancer brain metastasis^88^ with elevated levels present in samples of brain metastasis of breast cancer.^89^

Mass spectrometry analysis was confirmed via Western blot analysis of FeEVs, EVs and cell lysate samples derived from 4T1BR5-L2G cells, which all showed expression of Alix and Flotillin-1^70^ (Figure S2). Super resolution microscopy confirmed the presence of tetraspanins CD81 (magenta), CD9 (yellow), and CD63 (cyan) in samples of FeEVs derived from 4T1BR5-L2G cells (Figure S3). Aside from being typical EV markers, tetraspanins are also implicated in a number of processes.^90,91^ Although recently the tetraspanins CD63 and CD9 have been shown to not be required for EV uptake and content delivery,^92^ we did observe the tetraspanin CD81 and flotillin-1, both of which have been implicated in EV uptake.^90,91,93−96^

**Figure 2:**
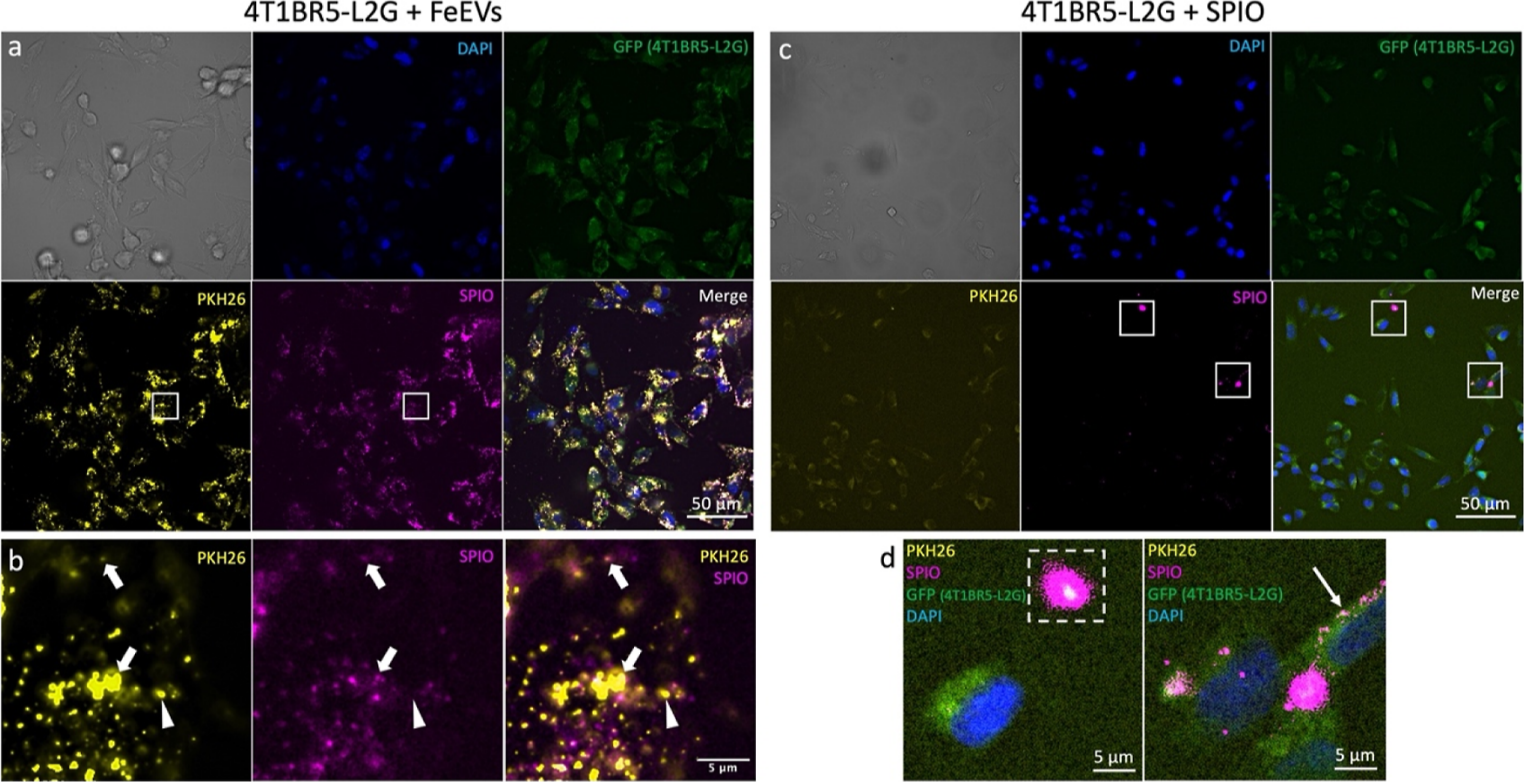
SPIO associated with EVs (FeEVs) accumulate more than SPIO alone in 4T1BR5-L2G cells in culture. After 24 h incubation, there was more SPIO in 4T1BR5-L2G cells when introduced as FeEVs (a) versus SPIO alone (c). The 4T1BR5-L2G cells are identified by blue (DAPI = nuclei) and green (GFP). The SPIO was tagged with a far-red fluorophore (magenta), and FeEVs or SPIO were dyed with PKH26 (yellow) to identify membranes. In the samples which were incubated with FeEVs (b, zoomed in from area identified in a), there are both regions with PKH26 and far-red SPIO fluorescence in the same spatial location (thick arrows, b), as well as PKH26 without far-red SPIO signal (arrowheads, b). In the samples that were incubated with SPIO alone, there was an extracellular far-red SPIO signal noted (dashed outline, d) and far-red SPIO appearing within the cells (thin arrow, d).

4T1 EVs have been studied and characterized previously,^97−99^ and there is evidence of preferential homing of EVs to a matched parent cancer cell line in vitro.^35,100,101^ The similarities in composition of the EV (i.e., lipid composition and surface receptors) and the matched parent cell line increase the propensity of fusion and internalization.^99^ Further, EVs do not exhibit any cytotoxicity when incubated with 4T1 cells in culture,^102^ highlighting their biocompatibility. Improved internalization of SPIO into 4T1BR5-L2G cells was seen when the SPIO was associated with EVs (FeEVs) versus SPIO alone (Figure 2). All cells, identified by DAPI (blue) and GFP (green), had PKH26 stained FeEVs (membrane stain, yellow, and SPIO far-red fluorescent, magenta), to varying extents (Figure 2a). In some instances, the PKH26 and SPIO fluorescence (yellow and magenta, respectively) were located in the same spatial location (thick arrow, Figure 2b), suggesting the association of PKH26-stained EVs and the SPIO. PKH26 without the SPIO signal was found in other locations (arrowhead, Figure 2b), suggesting the presence of unlabeled EVs. When cells were incubated with SPIO only (Figure 2c), minimal amounts of SPIO were found within the cells. Both extracellular SPIO was found (Figure 2d, dashed line) and SPIO without the PKH26 signal within cells (Figure 2d, thin arrow).

### Using MPI to Understand the Biodistribution of FeEVs and SPIOs

FeEVs and SPIOs were injected intravenously (iv) into healthy and 4T1 orthotopic tumor-bearing mice to understand the kinetics of FeEVs and SPIO biodistribution over time in vivo. When 4T1-derived FeEVs or equal amounts of iron were administered iv into healthy (FeEVs: 1.45 × 10^10^, SPIO: 8 μg) or tumor-bearing mice (FeEVs: 1.7 × 10^10^), MPI detected iron in the liver (Figure S4) but not in primary tumors. The high accumulation in the liver has resulted in challenges identifying and quantifying iron in regions of close proximity, by MPI.^103^ Here, this would result in difficulties visualizing the smaller amounts of iron that may have accumulated in the tumor.

### Primary Mammary Fat Pad Tumors Retain FeEVs More than Free Iron

**Figure 3:**
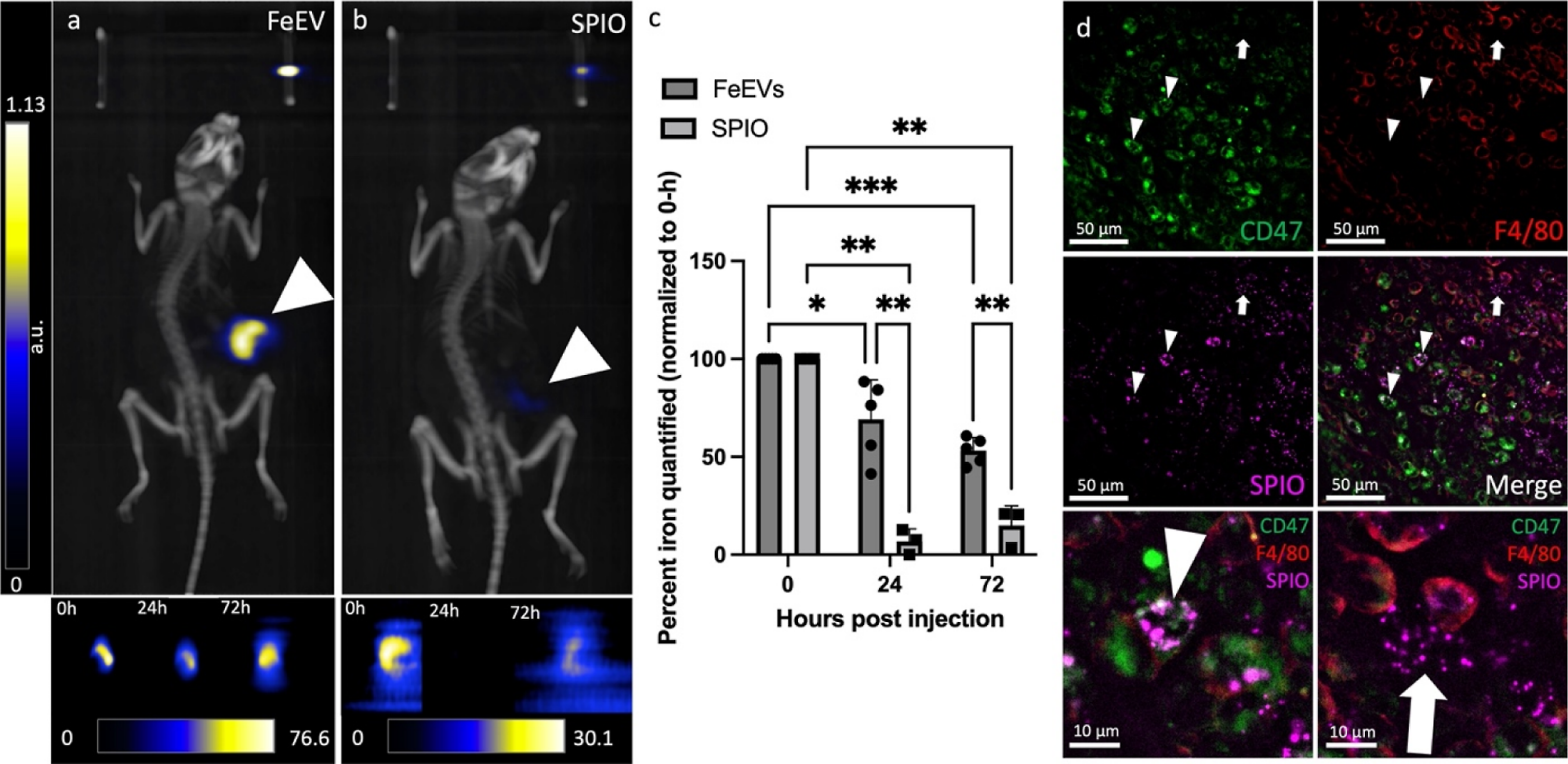
Increased retention of FeEVs in the location of the primary tumor compared to SPIO. Magnetic particle imaging (MPI) and microCT images (a,b, representative images, 72 h post-injection) were overlaid to localize iron signal after intratumoral injection of FeEVs (a) or SPIO (b) with cropped 2D MPI images of the tumor at 0, 24 and 72 h located below. Location of tumors is noted by white arrowhead. Tumor iron quantification (c) at 0, 24, and 72 h postinjection for FeEV- and SPIO-injected tumors (normalized to iron quantified at 0 h). Fluorescence microscopy (d) identifies SPIO (far-red, magenta), CD47 tumor cells (PE, green), and F4/80+ cells (AF647, red). SPIO and CD47+ cells in the same spatial location (arrowheads) and SPIO and F4/80+ cells within the same region (arrow). *p < 0.05, **p < 0.01, ***p < 0.0001.

FeEVs and SPIOs were injected intratumorally (it) into 4T1 orthotopic tumor-bearing mice to compare iron retention over time in vivo. The it injection of FeEVs (1.7 × 10^10^ FeEVs) or equal amount of iron delivered via SPIOs (9.6 μg) resulted in different retention dynamics (Figure 3). Localization of the iron was detected via MPI immediately after injection of FeEVs and SPIOs (Figure 3a,b). In tumors which received an FeEV injection, a higher percentage of iron was quantified from the injected dose (93.4%) immediately (0 h) after injection as compared to tumors injected with SPIO (63.6%, *p* = 0.009, Figure 3c). There was a significant decrease in the amount of iron detected 24 h (h) after the injection of FeEVs into tumors (69.2% of iron quantified at 0 h, *p* = 0.026), but the amount of iron detected at 72 h post-FeEV injection did not decrease further (53% of iron quantified at 0 h, *p* = 0.06). However, there was a large decrease in the amount of iron quantified 24 h after SPIO injection into the tumors (6.8% of iron detected at 0 h, *p* = 0.002), with no further difference in the amount of iron detected at 72 h postinjection (*p* = 0.373, compared to 24 h), with 14.9% of iron quantified at 0 h present. Immediately postinjection, significantly more iron detected in tumors injected with FeEVs suggests that there is rapid clearance of free SPIO from the tumor, whereas there was an initial retention of iron associated with EVs. At 24 h post-injection, there is a decrease in iron quantified in both FeEV and SPIO-treated groups. However, the tumors injected with FeEVs or SPIO had no significant difference in iron quantified at 24 or 72 h, suggesting the vast majority of SPIO clearance from the tumor occurs within the first 24 h of injection, while tumor retention is promoted in iron associated with EV membranes.

Histology of tumors excised at 72 h post-injection revealed the localization of FeEVs. No iron was detected in the mammary fat pad (MFP) tumors that had been injected with SPIOs, corresponding to the very low amount of iron detected by MPI. However, within the tumors injected with FeEVs, iron (magenta) was found inside CD47+ tumor cells (green; Figure 3d, white arrowhead) but not F4/80+ cells (red; Figure 3d, white arrow), indicating that cargos associated with FeEVs could be delivered to tumor cells. F4/80 serves as a common macrophage surface marker, although it can also be used to define dendritic cells.^104^ Further, 4T1 tumors have been shown to contain a high number of tumor-associated macrophages (TAMs),^105,106^ validating our findings. While CD47+ tumor cells showed SPIO uptake with a cell-like shape, F4/80+ cells did not appear to contain SPIO; rather, it was located near these cells. It is unlikely the tumor cells internalized SPIO alone, as SPIOs administered in vivo are typically only found in macrophages.^103,105,107^ This demonstrates that FeEVs can communicate or deliver cargo to tumor cells, which suggests the likely reason that the iron associated with EVs was detected by MPI in greater quantity and for longer than SPIOs.

EVs derived from 4T1 cells have been used previously in vivo, to identify localization to targeted metastatic sites (lungs, liver, and spine),^98^ and their targeted delivery of cargo to parental 4T1 tumors.^97^ These studies, along with our immunocytochemistry, suggest that the cellular origin has an influence on the localization of the EVs after administration in vivo.

### Association of Iron with EVs Allows Iron to Accumulate in the Heads of Mice with Brain Metastasis

**Figure 4:**
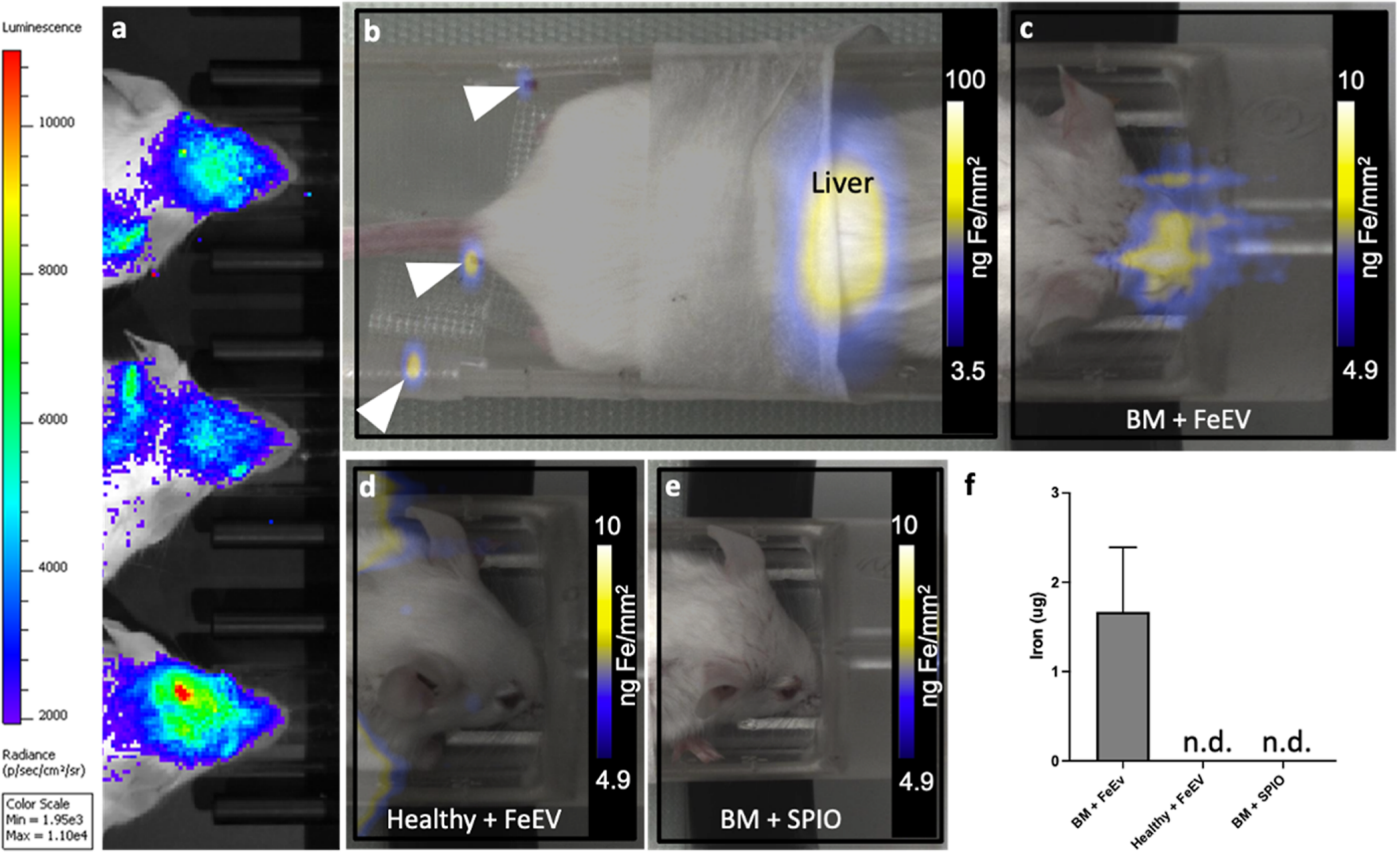
Magnetic particle imaging detects and quantifies iron in the heads of mice. BLI (a) was used to confirm brain metastasis in the heads of mice which received intracardiac administration of 4T1BR5-L2G cells. MPI and brightfield images were overlaid to localize MPI signal. MPI signal was located in the liver (representative image, b) in all groups. White arrowheads note the location of iron fiducials. MPI signal was only located in the head of mice with brain metastasis which were injected with FeEVs (c, BM + FeEV). There was no signal located in mice which did not have brain metastasis and received FeEVs (d, Healthy + FeEVs) or mice which had brain metastasis and received SPIO (e, BM + SPIO). MPI was used to quantify the iron in the heads of mice from each group (f). n.d. = not detected.

Experimental brain metastases were initiated by administering 4T1BR5-L2G cells into the left ventricle of the heart (intracardiac, ic). To confirm successful delivery, mice were imaged using BLI to confirm tumor cell localization in the brain (Figure 4a) and continued to observe the growth of brain metastases. Following ic administration of FeEVs (6.97 × 10^10^, average of 33.64 ± 5.44 μg Fe), the majority of iron (as determined by MPI signal) was found in the liver (Figure 4b) both in mice burdened with brain metastasis (93% of injected) or healthy controls (102% of injected). Thus, the liver still filters much of the FeEVs and SPIOs, despite injection into the left ventricle.

MPI signal due to the presence of iron was detected in the heads of 10/11 mice with brain metastases post-FeEV injection, with iron amounts averaging 1.67 ± 0.72 μg of Fe (Figure 4c), amounting to 4.96% of the injected dose. Furthermore, no MPI signal was detected in the heads of healthy mice ic injected with FeEVs, or in the heads of mice with brain metastases ic injected with SPIOs (Figure 4d,e). This demonstrated improved FeEV targeting to brain metastasis and delivery of cargo, implying that FeEVs have the potential for future diagnostic imaging and therapeutic targeting.

EVs have been used to deliver payloads to the brain in mice through intranasal administration^20,21,108−110^ and systemic injection.^19,25,26^ In addition to their ability to cross the BBB, EV use is favorable because of low immunogenicity, biodegradability, and their nontoxic nature. Further, they can carry cargo, such as iron particles in this study and others,^25,27,29,32,33,111,112^ as well as therapeutics.^18,19,21−24,26,109,110^ The mechanisms in which EVs cross the BBB is not fully understood; however, some possible routes which have been explored are micropinocytosis, clathrin-dependent endocytosis, and caveolae-dependent endocytosis.^16,17,113,114^

### Histology Reveals Iron Accumulation in Brains

**Figure 5:**
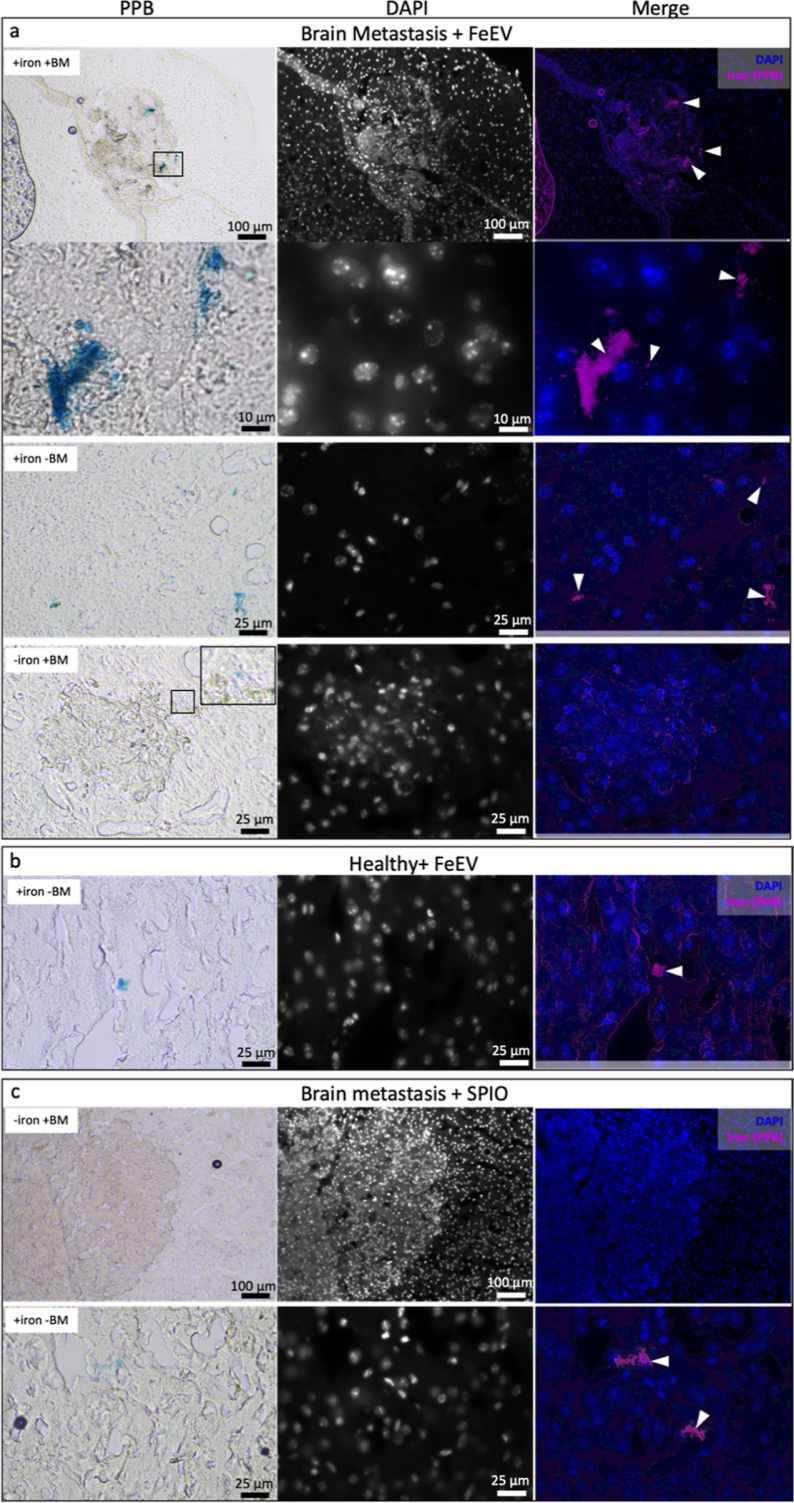
Histology locates iron present in brain sections. Sections were stained with PPB for iron (left column) and DAPI to visualize nuclei (middle column). Iron was pseudocolored magenta to overlay with DAPI (blue, right column). Sections from the group that had brain metastasis and received FeEVs (a) had regions where there was iron within metastasis, iron outside of metastasis, and metastasis not associated with iron. Mice that did not have metastasis and received FeEVs (b) did have some iron visualized. Mice which had metastasis and received iron (SPIO) had metastasis visualized without iron and iron within regions of no metastases.

Brain sections from FeEV- and SPIO-injected mice with brain metastases and FeEV-injected healthy mice were stained with Perls Prussian blue (PPB; blue = iron, pseudocolored magenta in the overlay) to visualize iron and DAPI to visualize nuclei. Within brain sections from mice with metastasis injected with FeEVs, we found iron associated with brain metastasis (as determined by changes in tissue architecture with increased and disorganized nuclei by DAPI),^115^ as well as within regions without an apparent brain metastasis (Figure 5a). There were also brain metastasis that did not have any iron associated with them (Figure 5a). In sections derived from brains of healthy mice injected with FeEVs, iron was found in lesser amounts (Figure 5b). Further, the group with brain metastasis that received SPIO also had iron found in the brain sections; however, no iron was found associated with brain metastasis (Figure 5c), and the iron identified was in lesser amounts. There was more apparent iron seen within the brain sections of mice with brain metastasis injected with FeEVs; the localization of the iron also appeared more intentional, either within the metastasis or surrounding it. Comparatively, iron in mice with brain metastasis injected with SPIOs and healthy mice injected with FeEVs were in lesser amounts, correlating with the undetectable iron levels in MPI.

Experimental brain metastasis models, including the 4T1BR5 model used in this study, have shown variable effects on the BTB. Although the BTB is impaired in the majority of these metastases, fewer than 10% exhibit leakiness which allows for enough drugs to enter to elicit therapeutic effects.^11,12^ BTB impairment could explain the presence of iron identified by PPB, with passive delivery of the iron, whereas an increase in iron accumulation when associated with EVs (FeEVs) can be considered an improvement in delivery, independent of BBB/BTB status. Therefore, there is still a need to develop ways to improve the delivery both when the BBB is intact or even when there is BTB heterogeneity in the tumors.

## Conclusions

Iron-labeled EVs have been imaged previously using MRI^26−28,31,32^ and MPI.^33^ Methods used to label EVs have been through direct labeling or indirect labeling where the parental cell is coincubated with the iron and is allowed to take up the nanoparticle. In this study, we found protamine sulfate and heparin increased parental cell labeling,^116^ resulting in increased FeEV loading and improved detection when used for MPI. TEM imaging of FeEVs shows that iron is associated with the EV membranes, and proteomics analysis confirms that the FeEVs contain proteins that are considered to be EV markers. In primary breast tumors in vivo, FeEVs from the breast cancer cells were retained for longer and in greater amounts as compared to iron nanoparticles alone, as demonstrated using MPI and confirmed by histological analysis of the tumor. Association with EV membranes also allowed SPIO nanoparticle delivery to the heads of mice when brain metastases were present. MPI could not detect iron in the heads of healthy mice injected with FeEVs, nor in mice with brain metastases injected with SPIO nanoparticles alone. We conclude that association with EV membranes allowed for the cargo, iron nanoparticles, to access metastatic sites across the BBB/BTB. In the future, they could act as a delivery vehicle for therapeutics associated with iron nanoparticles or other therapeutic agents.

## Methods

### Cell Culture

4T1fLuc2 cells (4T1L2; provided by Dr. Bryan Smith, MSU), 4T1BGL cells (provided by Dr. Michael Bachmann, MSU) and 4T1BR5-fLuc/GFP cells (4T1BR5-L2G; provided by Dr. Paula Foster, Western University) were maintained in incubators set at 37 °C and 5% CO_2_. Cells were cultured in RPMI + Glutamax with 10% fetal bovine serum (FBS). Cells were counted using the Trypan blue exclusion assay prior to in vitro or in vivo experiments.

### Iron Labeling of Cells

Three million cells were seeded in a 10 cm^2^ dish for iron labeling. Protamine sulfate (40 μg/mL) or heparin (2 U/mL) and 70 nm dextran-coated Synomag-D (MicroMod, Germany, cat #104-00-701 or cat #126-00-701 (far red fluorescence); 1 mg/mL Fe) were added to 2.5 mL of FBS-free media. Both tubes were well mixed before the protamine sulfate was added to the heparin and Synomag-D. Five milliliters of this mixture was added to each plate, and 3 to 6 h later, 5 mL of complete media was added. The cells were then incubated for 24 h post-addition of iron prior to FeEV isolation (below). The equivalent of 1 dish of FeEVs was used for iv biodistribution and primary tumor studies, and the equivalent of 2 dishes of FeEVs was used for brain metastasis studies.

### FeEV Isolation Via Differential Centrifugation

Iron-labeled cells were washed three times with 10 U/mL heparin and once with phosphate buffered saline (PBS), and replaced with media containing 10% EV-depleted FBS. Cells were incubated at 37 °C for 24 h to allow for EV production. Conditioned media was centrifuged at 600*g* for 10 min to remove any cells. The supernatant was then centrifuged at 2000*g* for 20 min to remove apoptotic bodies and cell debris. The remaining supernatant containing FeEVs was subsequently centrifuged at 20,000*g* for 1 h to concentrate FeEVs, which were then washed in PBS and further concentrated via centrifugation at 20,000*g* for 1 h, before resuspending in PBS.

### EV Isolation Via Differential Ultracentrifugation

Non iron-labeled 4T1 cells were seeded at a density of 3 × 10^6^ cells in a 10 cm dish. Twenty-four h after seeding, the cells were washed twice with PBS to remove traces of media and replaced with media containing 10% EV-depleted FBS. Cells were incubated at 37 °C for 24 h to allow for EV production. Conditioned media was centrifuged at 600*g* for 10 min to remove any cells and the supernatant was centrifuged at 2000*g* for 20 min to remove apoptotic bodies and cell debris. Supernatant containing non labeled EVs was removed and centrifuged at 100,000*g* for 90 min to concentrate non labeled EVs. These non labeled EVs were then washed with PBS and recentrifuged for purity before resuspension in PBS.

### FeEV or SPIO Uptake into 4T1BR5-L2G Cells in Culture

200,000 4T1BR5-L2G cells were seeded per well, in a two-well chambered slide (Nunc Lab-Tek II, cat #S6565) and incubated at 37 °C and 5% CO_2_ overnight. FeEVs from 3 × 10^6^ seeded 4T1BR5-L2G cells were collected as above. FeEVs or 20 μg of Synomag-D SPIO (far-red fluorescence) were resuspended in PKH26 membrane dye (Sigma-Aldrich, cat #MINI26), as per manufacturers suggestion. FeEVs and SPIO were washed twice with PBS prior to adding to 4T1BR5-L2G cells. Cells and FeEVs or SPIO were incubated at 37 °C and 5% CO_2_ for 24 h. Cells were then washed and fixed with 4% paraformaldehyde (PFA). The chamber was removed, and cells were coverslipped using Fluoromount-G mounting medium with DAPI (Invitrogen, cat #00-4959-52). Sections were imaged using a Leica DMi8 Thunder microscope equipped with a DFC9000 GTC sCMOS camera and LAS-X software (Leica, Wetzlar, Germany). Large volume computational clearing (LVCC) was performed on the images. Images were prepared using Fiji software.^117^

### Nanoparticle Tracking Analysis

FeEV particle size and concentration were measured by using a ZetaView Nanoparticle Tracking Analyzer (Particle Metrix, Germany). An average of 50–150 particles were read per frame as a quality control. The analysis parameters used were: max area: 1000, min area: 10, Min Brightness 22, with 11 frames read twice per sample.

### Transmission Electron Microscopy

EVs and FeEVs were visualized via TEM (JEOL 1400-Flash Transmission Electron Microscope, Japan Electron Optics Laboratory, Japan). Following fixation in 16% PFA, FeEVs were allowed to absorb on 200-mesh, carbon-coated Formvar copper grids for 20 min before fixation in 2.5% EM-grade glutaraldehyde in 0.1 M PBS for 15 min at room temperature. The grids were stained with 2% uranyl acetate for contrast and washed with EM-grade PBS and HPLC-grade water before imaging.

### Western Blotting

Cells were lysed in mRIPA lysis buffer [150 mM sodium chloride, 1.0% Triton X-100, 0.25% sodium deoxycholate (SDC), 50 mM Tris, pH 7.4] consisting of a protease inhibitor (Thermo Fisher, A32955) and phosphatase inhibitor (Thermo Fisher, A32957). The supernatant was used as cell lysates. Protein concentration of 4T1BR-L2G cell lysate, FeEVs, and EVs isolated using ExoQuick (System Biosciences, CA, USA) was determined using the Pierce BCA Protein Assay kit (Thermo Fisher, 23225) using BSA as a standard. The protein quantification curve was completed in replicate 3 times, and the unknowns were all replicated twice.

A total of 15 μg of protein was added per well, mixed with DI H_2_O and RunBlue LDS sample buffer (4×) (Expedeon, NXB31010). The cell lysate mixtures were heated at 70 °C for 10 min, while the FeEVs and EVs were not heated to avoid aggregation. The proteins were separated using Mini-PROTEAN TGX Stain-Free Precast gels (BioRad, 4568093) at 100 V for 80–90 min in the BioRad Mini-Protean Tetra system and transferred to a nitrocellulose membrane using the BioRad Trans-Blot Turbo Transfer System, running at 25 V for 30 min. The membrane was then blocked using 5% w/v nonfat dry milk in TBST for 1 h at room temperature and then incubated with primary antibody (anti-Alix, 1:5000; Protein Tech, 12422-1-AP or anti-Flotillin-1, 1:5000; Fisher Scientific, BDB610820) at 4 °C overnight. The membrane was then washed three times using TBST, and incubated in secondary antibody (Antirabbit HRP-linked, 1:2000) at room temperature for 1 h. The membrane was washed again three times with TBST, before the Pierce ECL Western Blotting Substrate kit (Thermo Fisher, 32209) was added. The proteins and ladder were then imaged using the ChemiDoc MP imaging system (Bio-Rad Laboratories, Inc.) using the autoexposure and chemiluminescence to observe the bands and 635 nm of light with autoexposure for visualization of the ladder.

### LC/MS/MS Analysis

Protein solutions were mixed with 100 mM Tris–HCl (pH 8.5) supplemented to 4% (w/v) SDC to 270 μL. Samples were reduced and alkylated by adding TCEP and chloroacetamide at 10 and 40 mM, respectively, and incubating for 5 min at 45 °C with shaking at 2000 rpm in an Eppendorf ThermoMixer C. Trypsin, in 50 mM ammonium bicarbonate, was added at a 1:50 ratio (w/w), and the mixture was incubated at 37 °C overnight with shaking at 1500 rpm in the Thermomixer. Final volume of each digest was ∼300 μL. After digestion, SDC was removed by a phase extraction. The samples were acidified to 1% TFA and subjected to C18 solid-phase cleanup using StageTips1 to remove salts.

An injection of 5 μL (∼600 ng) was automatically made using a Thermo (http://www.thermo.com) EASYnLC 1200 onto a Thermo Acclaim PepMap RSLC 0.1 mm × 20 mm C18 trapping column and washed for ∼5 min with buffer A. Bound peptides were then eluted over 35 min onto a Thermo Acclaim PepMap RSLC 0.075 mm × 500 mm resolving column with a gradient of 5% B to 40% B in 24 min, ramping to 90% B at 25 min and held at 90% B for the duration of the run (buffer A = 99.9% water/0.1% formic acid, buffer B = 80% acetonitrile/0.1% formic acid/19.9% water) at a constant flow rate of 300 nL/min. Column temperature was maintained at a constant temperature of 50 °C using an integrated column oven (PRSO-V2, Sonation GmbH, Biberach, Germany).

Eluted peptides were sprayed into a ThermoScientific Q-Exactive HF-X mass spectrometer (http://www.thermo.com) using a FlexSpray spray ion source. Survey scans were taken in the Orbi trap (60,000 resolutions, determined at *m*/*z* 200) and the top 15 ions in each survey scan are then subjected to automatic higher energy collision induced dissociation with fragment spectra acquired at 15,000 resolution.

Data analysis was performed as follows. The resulting MS/MS spectra were converted to peak lists using MaxQuant2, v1.6.3.4 (http://www.maxquant.org), and searched against a protein database containing all mouse sequences available from Uniprot (downloaded from http://www.uniprot.org, downloaded on 20221114) and appended with common laboratory contaminants using the Andromeda3 search algorithm, a part of the MaxQuant environment. The MaxQuant output was then analyzed using Scaffold, v5.1.2 (http://www.proteomesoftware.com) to probabilistically validate protein identifications. Assignments validated using the Scaffold 1% FDR confidence filter are considered true.

### Super Resolution Microscopy

Isolated FeEVs from 4T1BR5-L2G cells were analyzed using the ONI EV Profiler kit, a phosphatidylserine-based capture reagent applied to the EV chip capture surface. The EV sample was then applied and fixed to the surface with the ONI EV fixation buffer. Labeled antibodies against tetraspanins (CD81-CF647, CD63-CF568 and CD9-CF488A) were applied to the captured EVs, followed by another fixation step. dSTORM imaging buffer was applied to the samples and imaged on the ONI Nanoimager using the following dSTORM imaging conditions: 30 °C, 52° illumination angle (TIRF), and 30 ms exposure per frame. The following lasers were used, sequentially, in a 3000-frame light program: 1000 frames of each 640, 561, and 488 lasers. Analysis was performed using ONI’s cloud-based platform, CODI.

### In Vivo Studies

Six-week-old female Balb/C mice were purchased from Charles River Laboratories, and kept in the MSU animal facilities with approval from the MSU Institutional Animal Care and Use Committee. Mice which did not have tumors received FeEVs (from 4T1BGL; *n* = 2, 1.45 × 10^10^) or SPIO (Synomag-D far red, *n* = 2, 8.2 μg). Following iv administration, the mice were imaged using MPI and CT (described below) at 24 h, 48 h, and 7 d postinjection to observe the localization of iron.

Primary tumors were established by injecting 3 × 10^5^ 4T1L2 cells into the MFP and experiments were initiated 3 weeks after injection. A sample of FeEVs was imaged using MPI prior to injection to determine an estimate of iron present. 4T1L2-derived FeEVs (*n* = 5, 1.7 × 10^10^) or equal amount SPIO (Synomag-D, *n* = 3, 9.6 μg) were injected into the tumor (intratumoral; it) in 25 μL of PBS. Following it administration, mice were imaged with the standard 2D imaging mode in MPI and CT (described below). Following the final imaging time point, mice were sacrificed using 5% carbon dioxide, and underwent postmortem dissection to remove tumors.

Experimental brain metastasis were initiated in mice via intracardiac (ic) injection of 2 × 10^4^ 4T1BR5-L2G cells, resuspended in 85 μL of PBS mixed with 15 μL of ultrasound microbubbles (FUJIFILM VisualSonics, WA, USA). Mice were anesthetized (2% isofluorane in oxygen), followed by application of a depilatory to remove fur on their chest. Subcutaneous administration of ketoprofen (5 mg/kg) was used as an analgesic. The mice were placed supine, with their extremities secured, and the left ventricle of the heart was located followed by guidance of the needle and injection of the cell/microbubble mixture using ultrasound (Vevo 2100, Visualsonics). Mice were monitored for brain metastasis (relating to luminescence) using the IVIS Spectrum (PerkinElmer). One-hundred microliters of D-luciferin (PerkinElmer, CT, USA, cat #122799; 30 mg/mL) was injected ip 15 minutes prior to imaging, immediately following ic cell injection and imaging was performed every 48 h until FeEV administration. A sample of FeEVs was imaged using MPI prior to injection to determine an estimate of iron present. 4T1BR5-L2G-derived FeEVs (6.97 × 10^10^) or SPIO (30 or 36 μg) were injected ic 7–8 days following brain metastasis establishment. Mice were given analgesic (ketoprofen, 5 mg/kg) subcutaneously prior to beginning the procedure. Ultrasound was used (as described above) to administer FeEVs or SPIOs. Following ic injection, mice were imaged using MPI and CT (described below).

### In Vivo Imaging

Imaging was performed at the time points described using the following parameters. MPI was performed using the standard 3D and 2D imaging modes (FOV 12 × 6 × 6 cm, 5.7 T/m gradient, 1 (2D) or 21 (3D) projections, and 1 average). Mice were then transferred to a Quantum GX microCT scanner (PerkinElmer). Whole-body CT images were acquired using 3 × 8 s scans with the following parameters: 90 kV voltage, 88 μA amperage, 72 mm acquisition FOV, and 60 mm reconstruction FOV, resulting in 240 μm voxels. Standards of known iron amount were placed in the MPI bed to aid in coregistration of μCT and MPI scans.

### Image Analysis

MPI data sets were visualized and analyzed using Horos imaging software (Horos is a free and open source code software program that is distributed free of charge under the LGPL license at Horosproject.org and sponsored by Nimble Co LLC d/b/a Purview in Annapolis, MD, USA).

MPI quantification was performed on 2D images. Signal threshold was chosen by selecting an area of background from the image that did not have any iron present (primary tumor: adjacent gut signal; brain: signal outside of the head). 3× standard deviation of the background was set as a lower threshold to capture signal above this value using thresholding in Horos. For primary tumor quantification, the mean signal of the gut (minus the noise from a blank image) was subtracted from the mean signal from the tumor to account for any signal that was added to the tumor. In instances where the signal was low (i.e., SPIO injection, 72 h), if the thresholding spread to include other regions (i.e., the iron fiducials), it was removed manually. Iron signal in FeEV pellets, the primary tumor, or the brain of mice after injection of FeEV, EV, or SPIO were determined as described above, with total MPI signal calculated by mean signal x area. Mass of iron in the FeEV samples and measurements from NTA (above) were used to determine the amount of iron per FeEV.

Iron amount was determined using calibration lines. Different amounts of iron were imaged with MPI using imaging sequences to establish a reference curve for each scan type utilized: standard and high sensitivity 2D or standard 3D. A simple linear regression was performed to find the slope of the data (m) using best-fit values (*y* = known iron content and *x* = MPI signal) with the *x*,*y* intercept set to 0. The equation *y* = *mx* allowed for quantification of the iron content in FeEV pellets and in vivo by substituting the total MPI signal (*x*) from the ROI into the equation to solve for iron (*y*).

Iron concentration values displayed on scale bars were determined by plotting the mean signal (*x*) and iron/mm^2^ or iron/mm^3^ (*y*, based on 2D or 3D data sets) from the iron amounts used for the calibration lines. Concentration of iron was solved for by inputting mean signal (*x*) and solving for *y*.

### Histology

Brains and isolated primary tumors were fixed overnight in 4% PFA followed by cryopreservation through serial submersion in 10, 20, and 30% sucrose for 24 h each. Samples were then placed in a bed of OCT, before being flash frozen in a mixture of dry ice and ethanol. The frozen samples were stored at −20 °C in preparation for tissue sectioning using a cryostat (Leica CM3050 S, 10-μm thickness).

For primary tumors, tissue sections with far-red expression from the iron nanoparticles, as determined by screening, were washed with PBS for 5 min before being added to 0.3% triton X-100 in PBS and incubated for 45 min. Slides were then incubated in blocking buffer (5% goat serum and 0.3% triton X-100 in PBS) for 60 min. Anti-CD47-PE (3 μg/mL; Biolegend, cat #127507) and F4/80 Monoclonal Antibody (1:200; Thermo Fisher, cat #14-480185) were then added to the slides overnight at 4 °C followed by washing with PBS. Sections were then incubated with a secondary Goat anti-Rat IgG antibody, AF647 (1:500, Thermo Fisher, cat #A-21247) for 2 h at room temperature followed by washing with PBS. Sections were imaged using a Leica DMi8 Thunder microscope equipped with a DFC9000 GTC sCMOS camera and LAS-X software (Leica, Wetzlar, Germany). LVCC was performed on the images. Images were prepared using Fiji software.^117^

Brain sections were washed with PBS for 5 min followed by PPB staining to visualize iron. DAPI mounting media (Fluoromount-G, Invitrogen) was used to visualize nuclei. Sections were imaged using a Nikon Eclipse Ci microscope equipped with a Nikon DS-Fi3 high-definition camera (Nikon Instruments Inc. Tokyo, Japan) for color and brightfield acquisition, CoolSNAP DYNO (Photometrics, AZ, USA) for fluorescent imaging, and NIS elements BR 5.21.02 software (Nikon). Images were prepared using Fiji software.^117^ PPB staining (blue) was pseudocolored magenta for overlay with DAPI.

### Statistical Analysis

Statistical analyses were performed using Prism software (10.1.1, GraphPad Inc., CA, USA). A two-way repeated measure ANOVA with uncorrected Fisher’s LSD was used to compare differences in EV associated proteins between FeEVs and EVs derived from 4T1L2 or 4T1BR5-L2G cells. A two-way repeated measure ANOVA with uncorrected Fisher’s LSD was used to compare differences in iron quantification in primary tumors between those with that received FeEVs or SPIO, and between 0, 24, 48 and 72 h. Data are expressed as mean ± standard deviation; *p* < 0.05 was considered a significant finding.

## Supporting Information Available

Representative FeEV characteristics and iron content for FeEVs injected in vivo, MPI of Synomag-D SPIO and FeEV pellet, Western blot analysis of FeEV, EV and cell lysate for 4T1BR5-L2G, super resolution microscopy of FeEVs, in vivo biodistribution of SPIO or FeEVs into healthy mice (PDF)
