# Supplementary material for: Magnetic Particle Imaging Reveals that Iron-Labeled Extracellular Vesicles Accumulate in Brains of Mice with Metastases
Source: ACS Appl Mater Interfaces. 2024 Jun 11;16(24):30860–73. doi: 10.1021/acsami.4c04920 (PMC11194773; doi:10.1021/acsami.4c04920)
Supplement: Supplementary file 1 — am4c04920_si_001.pdf [file am4c04920_si_001.pdf]

Supporting Information

Magnetic particle imaging reveals that iron-labeled extracellular vesicles accumulate in brains of mice with metastases

Victoria A Toomajian,[1,2] Anthony Tundo[1], Evran E Ural,[1,2] Emily M Greeson,[1,3] Christopher H Contag,[1,2,3] Ashley V Makela[1*]

[1]Institute for Quantitative Health Science and Engineering, Michigan State University, East Lansing, MI 48824
[2]Department of Biomedical Engineering, Michigan State University, East Lansing, MI 48824
[3]Department of Microbiology, Genetics & Immunology Michigan State University, East Lansing, MI 48824
*Corresponding author: Ashley V Makela (makelaas@msu.edu)

	Average peak size	Number of FeEVs administered	Total iron administered (ug)	Iron (ug)/EV	Seeded cell density
Healthy + 4T1BGL FeEVs	93.1 nm (n=3)	$1.45x10^{10}$	6.13 ug	$4.24x10^{-10}$	$3x10^{6}$
Primary tumors + 4T1L2 FeEVs	95.6 nm (n=3)	$1.70x10^{10}$	9.63 ug	$5.66x10^{-10}$	$3x10^{6}$
Brain metastasis + 4T1BR5-L2G FeEVs	101.1 nm (n=3)	$6.97x10^{10}$	35.63 ug	$5.11x10^{-10}$	$6x10^{6}$

Table S1. Representative FeEV characteristics and iron content for FeEVs injected into non-tumor bearing (healthy) mice, mice with primary tumors and mice with brain metastasis.

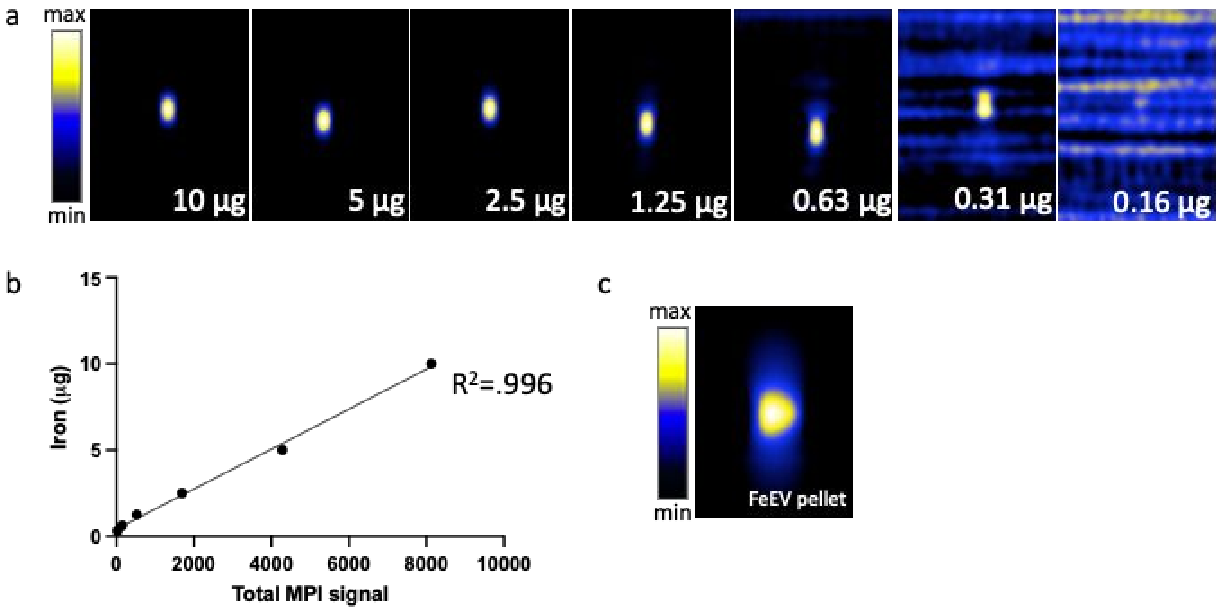

Figure S1. Magnetic Particle Imaging of Synomag-D SPIO and FeEV pellet. Different amounts of Synomag-D (0.16 μg – 10 μg) in 1 μl volumes imaged by MPI (**a**). There is a linear relationship between known iron amount (μg, y) and total MPI signal (x) (**b**). Representative FeEV pellet imaged by MPI prior to *in vivo* administration (**c**).

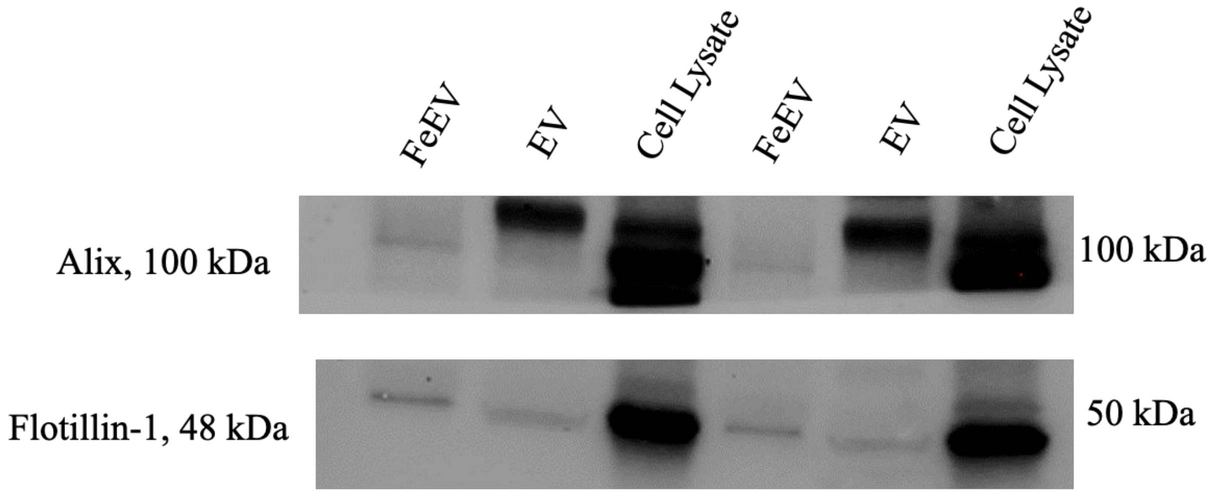

Figure S2. Western blot analysis of 4T1BR5-L2G-derived iron-labeled EVs (FeEVs), EVs and cell lysate. FeEVs, EVs and cell lysate from 4T1BR5-L2G cells contain Alix (upper row) and Flotillin-1 (lower row). Repeated data is a technical replicate.

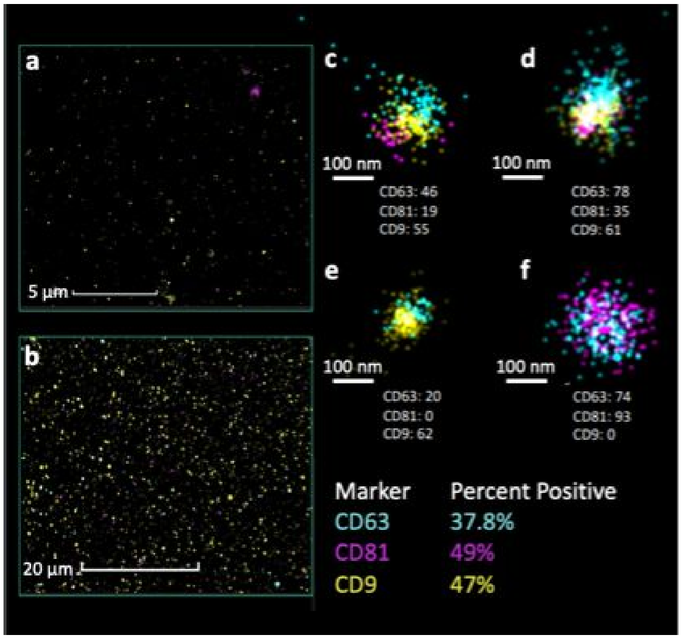

Figure S3. Super resolution microscopy of iron-labeled extracellular vesicles (FeEVs) derived from 4T1BR5-L2G cells. The sample was surface stained with anti-CD63 (CF568, cyan), anti-CD81 (CF647, magenta) and anti-CD9 (CF488A, yellow) antibodies. dSTORM imaging of sample at different magnifications (**a,b**). Four different FeEVs are shown (**c-f**), with different compositions of markers.

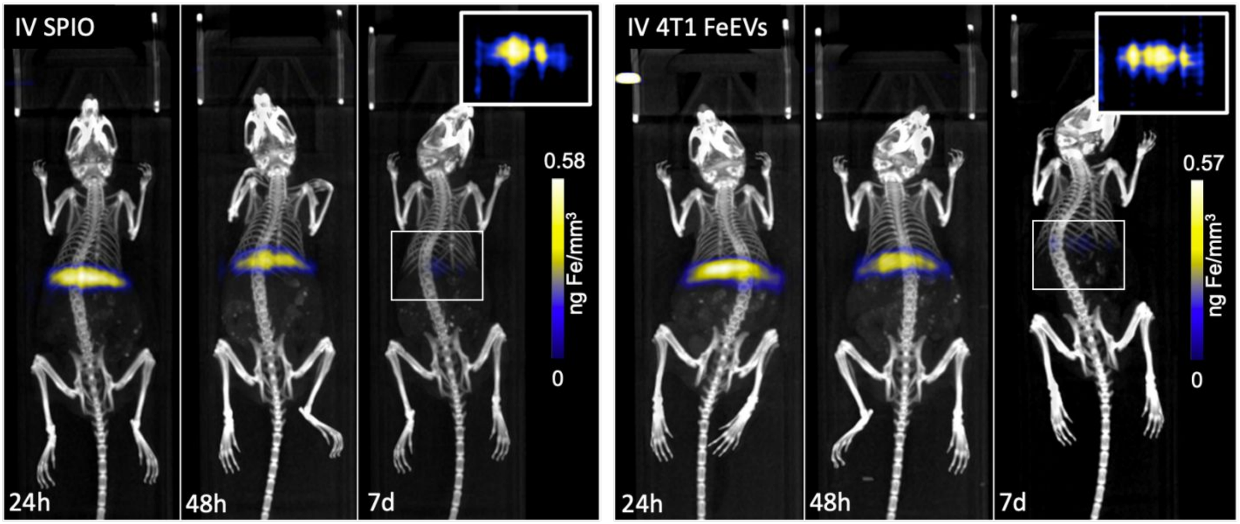

Figure S4. *In vivo* biodistribution of SPIO or 4T1 iron-labeled EVs (FeEVs) in non-tumor bearing mice. SPIO (left) or 4T1-derived FeEVs (right) were administered intravenous into non-tumor bearing mice. Magnetic particle imaging (MPI) and CT were performed at 24-hours (h), 48-h and 7-days (d); images are overlays of the MPI and CT images with intensity scale consistent longitudinally. Inset, top right is full dynamic range of signal from the liver at 7-d post injection.
